# Exploring biomechanical differences between brain-first and body-first Parkinson’s disease subtypes using shear wave elastography: a pilot cross-sectional study

**DOI:** 10.3389/fnagi.2026.1706502

**Published:** 2026-02-02

**Authors:** Shuangshuang Dong, Bo Shen, Yihong Song, Haiying Zhang, Yang Zhao, Yunyang Chen, Xu Jiang, Jun Zhu, Yang Pan, Ben Liu, Li Zhang

**Affiliations:** 1 Department of Neurology, The First Affiliated Hospital of Kangda College of Nanjing Medical University/The First People’s Hospital of Lianyungang, Lianyungang, China; 2 Department of Clinical Medicine, Kangda College of Nanjing Medical University, Lianyungang, China; 3 Department of Geriatric Neurology, Affiliated Brain Hospital of Nanjing Medical University, Nanjing, China; 4 Department of Physical Diagnosis, Affiliated Brain Hospital of Nanjing Medical University, Nanjing, China; 5 Medical School, Nanjing University, Nanjing, China

**Keywords:** body-first, brain-first, Parkinson’s disease, shear wave elastography, ultrasound

## Abstract

**Objective:**

This exploratory study aimed to investigate whether ultrasound shear wave elastography (SWE) could quantify rigidity in Parkinson’s disease (PD) and assess potential biomechanical differences between the brain-first and body-first subtypes.

**Methods:**

Shear wave elastography was used to measure the Young’s modulus (YM) and shear wave velocity (SWV) of the biceps brachii in 70 healthy controls (HCs) and 102 patients with PD (40 classified as body-first, 62 classified as brain-first based on RBDSQ criteria). Group comparisons were conducted using *t*-tests or ANOVA, correlations were assessed via Pearson or Spearman tests, and diagnostic performance was evaluated using receiver operating characteristic (ROC) curve analysis.

**Results:**

Young’s modulus and SWV values were significantly higher in PD patients than in HCs (all *p* < 0.001). The body-first group exhibited significantly greater YM and SWV than the brain-first group (all *p* < 0.001). Both SWE parameters showed significant correlations with several clinical scores. Clinically, compared to the brain-first group, the body-first group presented with a higher burden of non-motor symptoms, greater rigidity and axial impairment, but less tremor (all *p* < 0.05). For discriminating PD from HCs, the area under the curve (AUC) was 0.844 for YM and 0.845 for SWV. For exploratory differentiation between body-first and brain-first groups, the AUC was 0.730 for YM and 0.705 for SWV.

**Conclusion:**

Shear wave elastography is a sensitive tool for quantifying rigidity in PD. This pilot study provides the first evidence of a significant difference in muscle biomechanical properties between PD subgroups classified according to current clinical criteria. While SWE shows promise as a supplementary biomarker, its integration with established subtyping markers (e.g., polysomnography, MIBG scintigraphy) in future multimodal and longitudinal studies is essential to determine its role in clinical subtyping.

## 1 Introduction

Parkinson’s disease (PD) is a common neurodegenerative disorder, for which aging represents the primary risk factor (Chhetri et al., 2023; Hou et al., 2019). Given the ongoing global population aging, the worldwide prevalence of PD is anticipated to increase. The disease is characterized by a constellation of motor symptoms, including rigidity, tremor, bradykinesia, and postural instability (Leite Silva et al., 2023; Yin et al., 2021; Burtscher et al., 2024). Among these, rigidity–defined as increased resistance to passive muscle stretch–reflects underlying abnormalities in muscle extensibility and compliance. Clinically, rigidity severity in PD is typically rated using tools such as the Hoehn-Yahr (H-Y) stage, the Unified Parkinson’s Disease Rating Scale (UPDRS), or the Modified Ashworth Scale (MAS) (Richards et al., 1994; Meseguer-Henarejos et al., 2018). However, these approaches are largely subjective, relying on clinician judgment, and lack quantitative precision. Magnetic resonance elastography (MRE) offers an objective alternative (Smith et al., 2023; Kennedy et al., 2020), yet it is hindered by high cost, long acquisition times, and the need for patients to remain still–a particular challenge in PD.

Shear wave elastography (SWE) is a noninvasive, convenient ultrasound-based technique that has gained broad acceptance for quantifying tissue elasticity and stiffness (Zhu et al., 2024). It operates by generating shear waves via an acoustic push pulse; these waves propagate through tissue, and their velocity is directly related to tissue stiffness. Tissue stiffness is quantified by measuring the shear wave velocity (SWV), which correlates positively with tissue hardness (Yin et al., 2021; Ding et al., 2021; Jiang et al., 2023). Since SWV reliably reflects biomechanical properties across physiological and pathological states, it holds considerable promise for assessing tissue stiffness. Young’s modulus (YM), another key parameter, is derived from SWV using the formula *E* = 3ρ × v^2^ (kPa), where ρ represents soft tissue density and v denotes SWV (Hug et al., 2015). Owing to these attributes, SWE has become a well-established diagnostic tool in hepatology, thyroidology, breast imaging, and urology (Gürün et al., 2021; Kolb et al., 2021; Bang et al., 2021; Gurun et al., 2021; Liu et al., 2021). Although musculoskeletal applications of SWE are more recent, the field is evolving rapidly (Cipriano et al., 2022; Zhu et al., 2024). Nevertheless, studies employing SWE to evaluate muscle stiffness in PD remain scarce. Several reports have demonstrated that PD patients exhibit elevated YM or SWV values compared with healthy individuals (Ding et al., 2021, 2024; Du et al., 2016).

Emerging hypotheses suggest that PD can be stratified into two distinct subtypes based on the initial site of α-synuclein (α-syn) pathology: brain-first and body-first PD (Borghammer and Van Den Berge, 2019). Notably, the presence of isolated rapid eye movement sleep behavior disorder (RBD) preceding the onset of parkinsonism strongly indicates the body-first subtype (Horsager et al., 2020, 2022). RBD is believed to originate from dysfunction in specific pontine regions, such as the magnocellular reticular nucleus and subcoeruleus area (Shen et al., 2020). Advanced neuroimaging techniques, including positron emission tomography (PET), ^123^I-MIBG myocardial scintigraphy, and striatal dopaminergic imaging, have been employed to differentiate these subtypes (Horsager et al., 2020; Park et al., 2024; Knudsen et al., 2021). However, their clinical utility is constrained by high cost, technical complexity, and substantial patient cooperation requirements, underscoring the need for additional, more accessible quantitative tools that may complement these established methods.

Differentiating between brain-first and body-first PD carries important clinical implications, as the subtypes differ in progression rates, non-motor symptom burden, and therapeutic response. Specifically, the body-first subtype is associated with a greater burden of non-motor symptoms–particularly autonomic dysfunction that is often refractory to levodopa (Horsager et al., 2022; Dong et al., 2024)–and may exhibit more rapid motor decline (Xu et al., 2024).

Given that the body-first subtype is characterized by earlier and more severe involvement of brainstem pathways (Horsager et al., 2022; Shen et al., 2020), and that rigidity–a core PD motor feature–arises from disordered brainstem circuitry and its descending projections to the spinal cord (Asci et al., 2023; Zampogna et al., 2024), we hypothesized that rigidity would be more pronounced in the body-first subtype. This could potentially manifest as quantifiable differences in muscle biomechanical properties. Although SWE represents a promising objective tool for rigidity assessment in PD (Ding et al., 2021, 2024; Du et al., 2016), no study to date has applied SWE to explore potential differences between brain-first and body-first PD subtypes. Therefore, as an exploratory investigation, we aimed to: (1) compare SWE-derived measurements of the biceps brachii in PD patients and healthy controls (HCs), and (2) examine whether these biomechanical metrics showed differences between PD patient groups classified according to current clinical subtyping criteria.

## 2 Materials and methods

### 2.1 Study design and participants

Participants were recruited from the Department of Geriatric Neurology at Nanjing Brain Hospital, China, between July 2023 and October 2024. Patients with PD were diagnosed by a neurologist according to the 2015 Movement Disorder Society clinical diagnostic criteria and were at H–Y stages I–III. The healthy control (HC) group comprised individuals with no diagnosis of PD or other neurological disorders. Exclusion criteria for all participants included: secondary parkinsonism, diabetes, schizophrenia, demyelinating diseases, amyotrophic lateral sclerosis, peripheral neuropathy, hyperthyroidism, hypothyroidism, history of neuromuscular disorders, prior upper limb trauma or surgery, history of stroke, or significant cognitive impairment.

A thorough neurological examination was conducted to rule out motor dysfunction in the upper limbs. Due to practical constraints associated with polysomnography (PSG, the gold standard for diagnosing RBD), the RBD Screening Questionnaire (RBDSQ) was used. The RBDSQ is a validated instrument in which a score ≥ 5 indicates probable RBD (Li et al., 2017; Pagano et al., 2018). Further review of medical history and structured interviews regarding dream-enactment behaviors were performed to exclude other potential causes of RBD, such as medication side effects or alcohol use. For participants with probable RBD, the temporal relationship between the onset of dream-enactment behaviors and motor symptoms was specifically assessed. In this study, PD patients with probable RBD symptoms (RBDSQ ≥ 5) that clearly preceded motor symptom onset were classified as the body-first subtype. Patients with RBD onset after motor symptoms were not included in the current cohort, and those without RBD were classified as the brain-first subtype (Horsager et al., 2020, 2022).

The final sample included 172 participants: 102 patients with PD and 70 HCs. The PD group was further divided into 40 cases of body-first PD and 62 cases of brain-first PD.

To address possible misclassification bias arising from the use of RBDSQ instead of PSG, a sensitivity analysis was conducted. Participants with borderline RBDSQ scores (4 or 5), for whom RBD status was most uncertain, were excluded. The remaining cohort was re-classified into a “definite body-first” group (RBDSQ ≥ 6) and a “definite brain-first” group (RBDSQ ≤ 3). All clinical and SWE parameters were then re-compared across these groups to evaluate the robustness of the primary findings.

### 2.2 Clinical assessment

All assessments were carried out by experienced neurologists. PD patients were evaluated during their regular medication “on” phase, which included collection of medical history, clinical and neurological examination, and completion of PD-specific rating scales. HCs underwent a similar set of assessments, excluding those specific to PD.

The following instruments were administered: the UPDRS, the RBDSQ, the Non-Motor Symptoms Questionnaire (NMSQ), the Parkinson’s Disease Sleep Scale (PDSS), the Hamilton Depression Scale (HAMD), the Hamilton Anxiety Scale (HAMA), the Mini-Mental State Examination (MMSE), and the Parkinson’s Disease Questionnaire-39 (PDQ-39). Cognitive function was assessed in all participants using the MMSE. Disease severity in PD was measured with the UPDRS; motor function was evaluated with UPDRS Part III (UPDRS-III), which provides subscores for tremor, rigidity, bradykinesia, and axial/gait symptoms. Non-motor symptoms were quantified with the NMSQ, and quality of life was assessed with the PDQ-39. Depression and anxiety were rated using the HAMD and HAMA, respectively, while sleep quality was evaluated with the PDSS.

### 2.3 SWE examination

Shear wave elastography was performed by an experienced ultrasound diagnostician using a Supersonic Aixplore ultrasound system (GE LOGIQ E20, USA) equipped with SWE technology and an L4–15 linear array transducer (frequency range: 4–10 MHz). The biceps brachii was chosen as the examination site for the following reasons: (1) as a major superficial muscle, it provides excellent accessibility and reliability for SWE measurements; (2) it is clinically relevant for rigidity assessment in Parkinson’s disease; (3) its superficial location minimizes technical limitations associated with shear wave propagation in deeper tissues; (4) this choice aligns with previous SWE studies in PD, enabling valid cross-study comparisons (Du et al., 2016; Ding et al., 2021).

Parkinson’s disease patients were examined during their medication “on” phase and had followed their usual medication regimen on the day of testing. The sonographer was blinded to the clinical diagnoses and group assignments of the participants. SWE was performed on the dominant arm in HCs and on the more affected side in PD patients. Prior to SWE, conventional B-mode ultrasound was used to exclude structural abnormalities such as muscle tears, tumors, or hematomas.

Participants were positioned supine with the elbow fully extended and rested for 5 min before measurement. During SWE acquisition, the operator’s hand was stabilized using a bracket to maintain minimal probe pressure, thereby preventing artifactual overestimation of muscle stiffness due to excessive compression (Ding et al., 2021).

**FIGURE 1:**
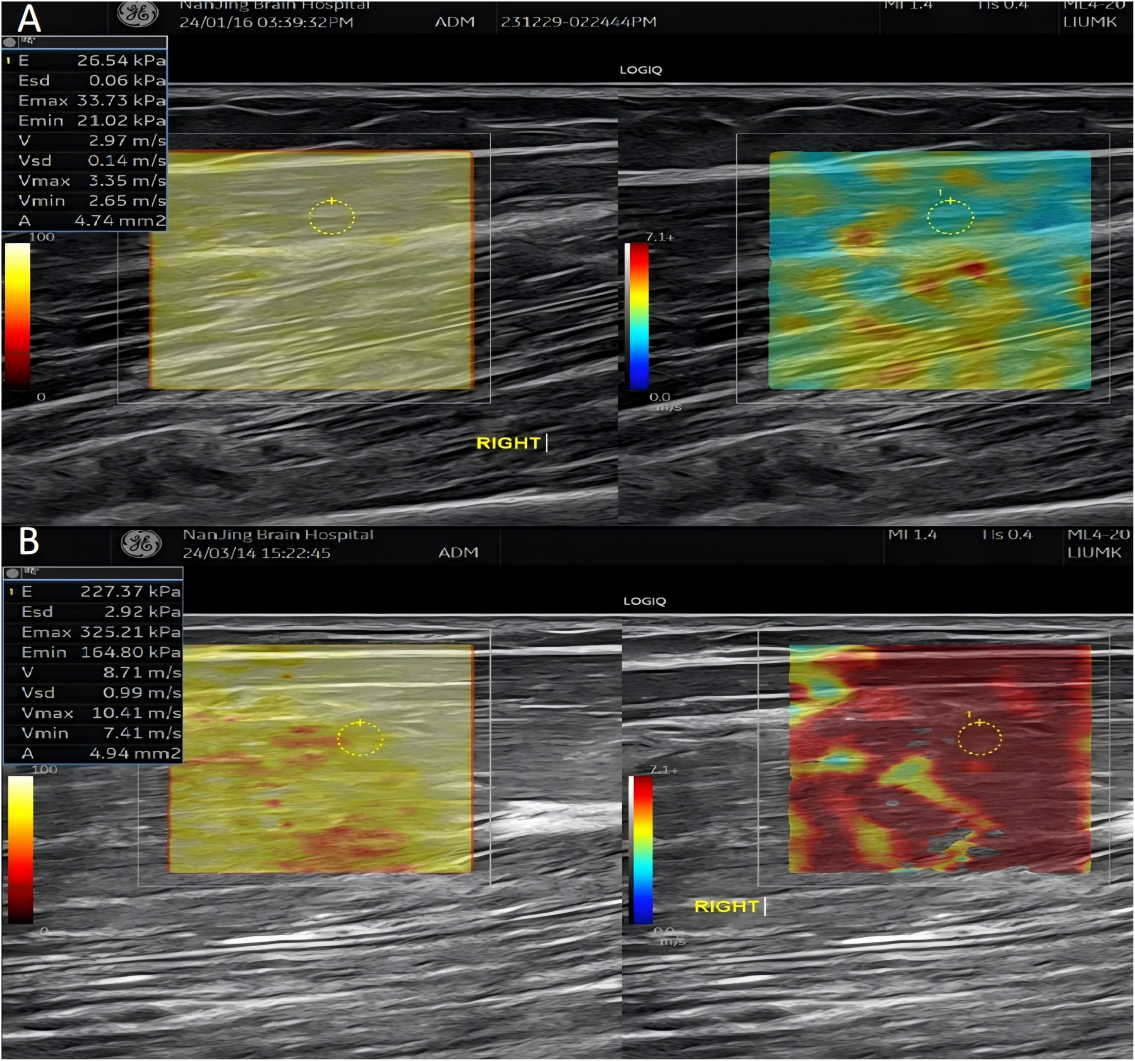
Representative shear wave elastography images of the biceps brachii. **(A)** Healthy control showing lower stiffness (blue-green map). **(B)** Parkinson’s disease patient showing elevated stiffness (red map). The region of interest (ROI) is indicated by a yellow circle.

The system was configured with a shear modulus range of 0–100 kPa, suitable for assessing resting muscle stiffness. A region of interest (ROI) was placed on the B-mode image, avoiding bones and large vessels. The probe was aligned parallel to the biceps muscle fibers to minimize anisotropy. Stiffness was measured within the biceps muscle at a depth of 10–20 mm beneath the skin, using a fixed 20 mm × 20 mm ROI positioned in the mid-portion of the muscle. After activating SWE mode, an acoustic radiation force impulse generated shear waves propagating through the ROI. Tissue stiffness was displayed on a color map (blue: soft; red: stiff). A quantitative analysis tool (Q-BOX) was used to measure values of YM and SWV within a central 2.5 mm diameter circle inside the ROI. Each muscle was measured twice, and the average values were used for statistical analysis (Figure 1).

To assess the reproducibility of SWE measurements, intra-rater reliability was evaluated. The same operator performed duplicate measurements on the same biceps brachii in a subset of 10 participants (5 HCs, 5 PD) on the same day. The intraclass correlation coefficient (ICC) for both YM and SWV exceeded 0.90, indicating excellent reliability.

### 2.4 Statistical analysis

Data were analyzed with SPSS version 25.0 (IBM Corp., USA) and visualized with GraphPad Prism version 8 (GraphPad Software, USA). Normality was evaluated using Q–Q plots and histograms. Normally distributed continuous variables are expressed as mean ± standard deviation (SD) and were compared with the two-sample *t*-test or ANOVA; non-normally distributed data are presented as median [interquartile range] and were compared with the Mann–Whitney U test or Kruskal–Wallis H test. Categorical variables are summarized as counts (percentages) and were compared with the chi-square test.

For the primary SWE parameters (YM and SWV), homogeneity of variances was checked with Levene’s test. Because variances were unequal for both parameters (Levene’s test *p* < 0.05), Welch’s ANOVA was employed for three-group comparisons. Where Welch’s ANOVA yielded a significant result (*p* < 0.05), *post hoc* pairwise comparisons were conducted using independent-samples *t*-tests without assuming equal variances.

To control for multiple comparisons, a two-tier Bonferroni correction was applied. For comparisons between PD and HC (Table 1), the correction factor was 2 (two SWE parameters), giving a significance threshold of *p* < 0.025. For three-group comparisons (HC, brain-first PD, body-first PD; Table 2), the correction factor was 6 (2 parameters × 3 pairwise comparisons), resulting in a threshold of *p* < 0.0083. Both raw and corrected *p*-values are reported where appropriate.

**TABLE 1:** Comparison of demographic, clinical characteristics, and SWE parameters between Parkinson’s disease patients and healthy controls.

Variable	PD (*n* = 102)	HC (*n* = 70)	*P*-values
Age (years)	67.40 ± 8.56	65.26 ± 8.64	0.110
Sex (male, *N*%)	53 (51.96)	28 (40.00)	0.123
BMI (kg/m^2^)	24.19 ± 3.78	23.61 ± 3.35	0.313
Hypertension (*N*%)	45 (44.12)	29 (41.43)	0.726
YM (KPa)	70.70 ± 36.70	33.33 ± 8.44	<0.001
SWV (m/s)	4.77 ± 1.25	3.30 ± 0.41	<0.001
RBDSQ	3.66 ± 3.36	1.31 ± 1.11	<0.001
MMSE	27.00 (23.00, 28.00)	28.00 (25.75, 29.25)	0.005

Data are expressed as mean ± SD, median (IQR), or count (%), as appropriate. SWE parameters were compared using a Bonferroni-corrected significance threshold of *p* < 0.025 for two comparisons. PD, Parkinson’s disease; HC, healthy control; SWE, shear wave elastography; BMI, body mass index; RBDSQ, Rapid Eye Movement sleep behavior disorder screening questionnaire; MMSE, Mini-Mental State Examination; YM, Young’s modulus; SWV, shear wave velocity.

**TABLE 2:** Comparison of demographic, clinical characteristics, and SWE parameters among healthy controls, brain-first PD, and body-first PD subgroups.

Variable	HC (*n* = 70)	PD (*n* = 102)		*P*-values
		Brain-first PD (*n* = 62)	Body-first PD (*n* = 40)	
**General information**				
Age (years)	65.26 ± 8.64	66.85 ± 8.93	68.25 ± 7.99	0.203
Sex (male, *N*%)	28 (40.00)	30 (48.39)	23 (57.50)	0.201
BMI (kg/m^2^)	23.61 ± 3.35	24.34 ± 3.96	23.96 ± 3.52	0.528
Hypertension (*N*%)	29 (41.43)	26 (41.94)	19 (47.50)	0.807
Disease duration (years)	–	3.00 (1.00, 4.00)	2.75 (1.00, 4.75)	0.997
**SWE ultrasound**				
YM (KPa)	33.33 ± 8.44	59.02 ± 28.56^a^*	88.80 ± 40.74^a^*^b^*	<0.001
SWV (m/s)	3.30 ± 0.41	4.44 ± 1.08^a^*	5.28 ± 1.33^a^*^b^*	<0.001
**Assessment scales**				
Hoehn and Yahr stage	–	2.38 ± 0.54	2.56 ± 0.60	0.109
UPDRS (total)	–	38.03 ± 15.32	44.58 ± 19.73	0.064
UPDRS-I	–	1.00 (0.00, 3.00)	2.00 (1.00, 3.00)^b^	0.041
UPDRS-II	–	10.16 ± 5.28	12.08 ± 7.38	0.132
UPDRS-III	–	25.79 ± 10.87	29.48 ± 12.34	0.117
Tremor score	–	4.23 ± 4.07	2.60 ± 2.99^b^	0.023
Rigidity score	–	4.87 ± 3.65	7.35 ± 3.95^b^	0.002
Bradykinesia score	–	10.85 ± 5.76	12.85 ± 7.07	0.123
Axial/Gait score	–	3.34 ± 1.53	4.08 ± 1.56^b^	0.022
UPDRS-IV	–	0.00 (0.00, 0.00)	0.00 (0.00, 0.00)	0.360
HAMD	–	5.89 ± 4.75	8.60 ± 7.01^b^	0.022
HAMA	–	4.25 ± 3.62	6.58 ± 5.13^b^	0.015
NMSQ	–	7.30 ± 4.55	9.75 ± 4.91^b^	0.012
PDSS	–	120.15 ± 21.24	110.15 ± 18.46^b^	0.017
RBDSQ	1.31 ± 1.11	1.29 ± 1.34	7.33 ± 1.95^a^^b^	<0.001
MMSE	28.00 (25.75, 29.25)	27.00 (23.00, 28.00)^a^	26.00 (22.25, 28.75)^a^	0.018
PDQ39	–	25.90 ± 20.37	35.67 ± 24.73^b^	0.033

Data are expressed as mean ± SD, median (IQR), or count (%). A Bonferroni correction was applied for SWE parameters (2 parameters × 3 comparisons; significance threshold: *p* < 0.0083). Superscripts indicate pairwise differences:

a: *p* < 0.05 vs. HC;

a*: *p* < 0.0083 vs. HC after Bonferroni correction;

b: *p* < 0.05 vs. brain-first PD;

b*: *p* < 0.0083 vs. brain-first PD after Bonferroni correction. PD, Parkinson’s disease; HC, healthy control; SWE, shear wave elastography; BMI, body mass index; RBDSQ, Rapid Eye Movement sleep behavior disorder screening questionnaire; MMSE, Mini-Mental State Examination; YM, Young’s modulus; SWV, shear wave velocity; UPDRS, Unified Parkinson’s Disease Rating Scale; HAMD, Hamilton Depression Scale; HAMA, Hamilton Anxiety Scale; NMSQ, Non-Motor Symptoms Questionnaire; PDSS, Parkinson’s Disease Sleep Scale; PDQ-39, Parkinson’s Disease Questionnaire-39.

Correlations between SWE parameters (YM, SWV) and participant characteristics (e.g., age, sex, BMI, hypertension, disease duration, and PD-related scale scores) were examined using Pearson or Spearman correlation analysis as appropriate.

Exploratory diagnostic performance of YM and SWV for identifying PD and for differentiating subtypes was assessed with receiver operating characteristic (ROC) curve analysis, using the area under the curve (AUC) as a measure of accuracy.

## 3 Results

### 3.1 Study population

The demographic and clinical characteristics of the study population are presented in Tables 1, 2. The PD group and the HC group were well matched in sex, age, body mass index (BMI), and prevalence of hypertension, with no significant between-group differences. Disease duration did not differ significantly between the body-first and brain-first PD subgroups.

### 3.2 Clinical assessment scales

Significant differences between PD patients and HCs were observed on multiple clinical scales (Table 1). The PD group showed significantly higher RBDSQ scores (*p* < 0.001), indicating more severe RBD symptoms, and lower MMSE scores (*p* = 0.005), suggesting greater cognitive impairment.

In comparisons between PD subtypes, the body-first group scored significantly higher than the brain-first group on UPDRS Part I (*p* = 0.041), rigidity (*p* = 0.002), and axial/gait symptoms (*p* = 0.022), while tremor scores were significantly lower (*p* = 0.023). The body-first subgroup also exhibited more severe non-motor symptoms and poorer quality of life, as reflected in higher scores on the HAMD (*p* = 0.022), HAMA (*p* = 0.015), NMSQ (*p* = 0.012), RBDSQ (*p* < 0.001), and PDQ-39 (*p* = 0.033). In addition, body-first PD patients had significantly lower PDSS scores (*p* = 0.017), indicative of worse sleep quality. No significant differences were found between the PD subgroups in total UPDRS score, UPDRS-II, UPDRS-III, UPDRS-IV, H–Y stage, bradykinesia, or MMSE scores (all *p* > 0.05; Table 2).

### 3.3 SWE ultrasound results

Shear wave elastography measurements demonstrated excellent intra-rater reliability, with ICC for both YM and SWV exceeding 0.90 in a reliability subset (*n* = 10).

Young’s modulus and SWV values were significantly higher in the PD group than in the HC group (all raw *p* < 0.001; all Bonferroni-corrected *p* < 0.001; Table 1). Levene’s test indicated unequal variances for both YM and SWV in the three-group comparisons (both *p* < 0.05). Welch’s ANOVA revealed significant overall differences among the three groups for YM (*p* < 0.001) and SWV (*p* < 0.001). *Post hoc* pairwise comparisons with Bonferroni correction (significance threshold *p* < 0.0083) showed that the body-first PD subgroup had significantly elevated values compared with both the brain-first PD group and the HCs: YM (body-first vs. Brain-first: corrected *p* < 0.001; body-first vs. HC: corrected *p* < 0.001) and SWV (body-first vs. Brain-first: corrected *p* < 0.001; body-first vs. HC: corrected *p* < 0.001). The brain-first PD subgroup also displayed significantly higher values than HCs for both SWE parameters (all corrected *p* < 0.001; Table 2).

**FIGURE 2:**
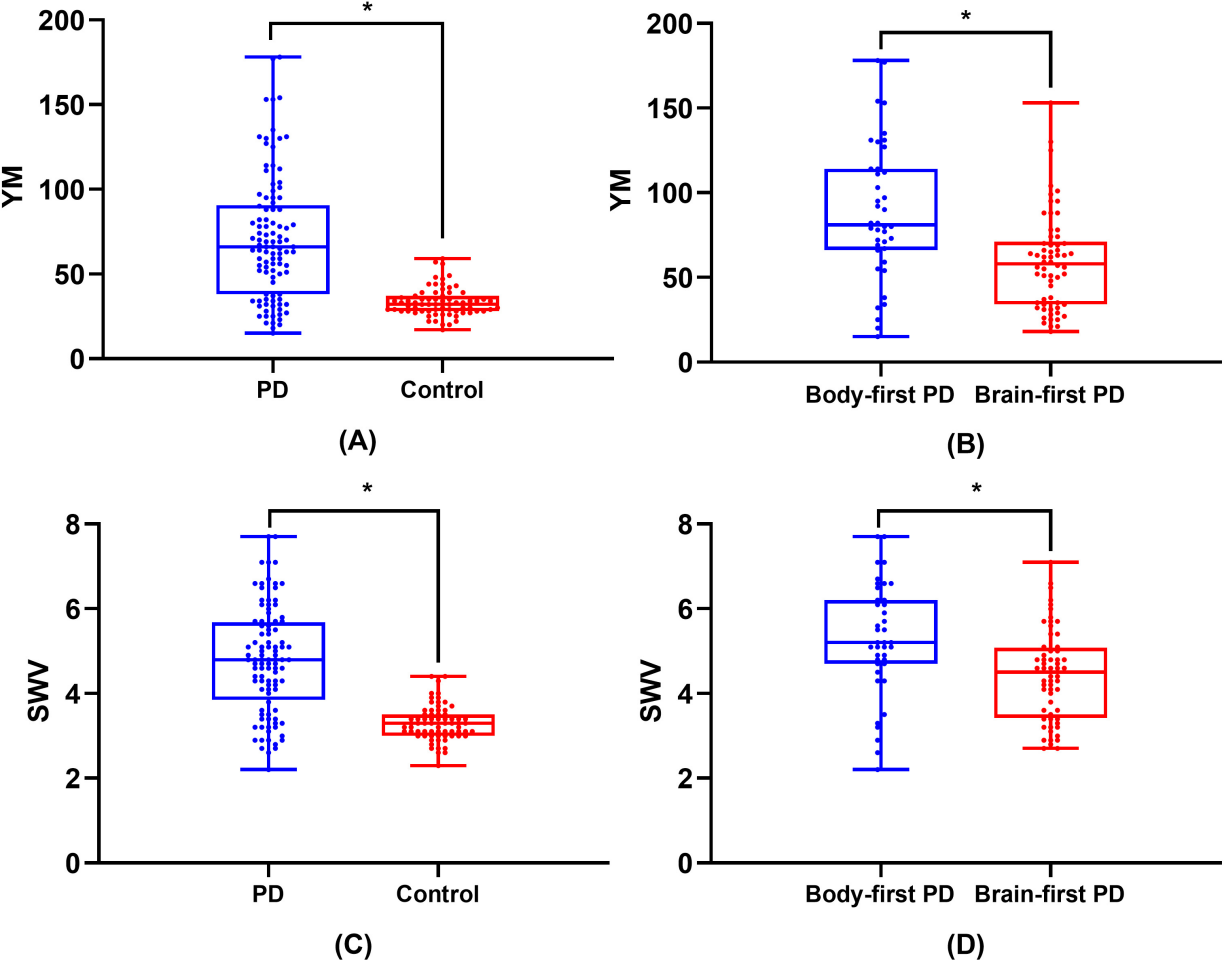
Box plots of SWE parameters across groups. **(A,C)** Young’s modulus (YM) and shear wave velocity (SWV) in healthy controls (HC) and all Parkinson’s disease (PD) patients. **(B,D)** YM and SWV in HC, brain-first PD, and body-first PD subgroups. Boxes represent interquartile range, lines indicate medians, whiskers show data ranges. *P < 0.001.

Box plots illustrating the distributions of YM and SWV across HCs, the overall PD group, and PD subgroups are shown in Figure 2. The HC group exhibited more compact distributions, indicating lower variability, together with lower median YM and SWV values compared with the PD group. The minimal overlap between the HC and PD groups suggests strong discriminative capacity of the SWE metrics. Within the PD subgroups, the body-first group showed higher median values than the brain-first group.

### 3.4 Relationship between SWE parameters and participant characteristics

Correlation analyses were conducted for both SWE parameters. YM showed a moderate positive correlation with the rigidity score (*r* = 0.456, *p* < 0.001). Weak positive correlations were observed with the RBDSQ score (*r* = 0.335, *p* < 0.001), UPDRS total score (*r* = 0.208, *p* = 0.037), UPDRS-III (*r* = 0.224, *p* = 0.024), and male sex (*r* = 0.199, *p* = 0.045). A weak negative correlation was found with BMI (*r* = −0.304, *p* = 0.002).

A parallel analysis for SWV revealed a highly consistent pattern of associations. SWV was significantly correlated with the rigidity score (*r* = 0.417, *p* < 0.001), RBDSQ score (*r* = 0.274, *p* = 0.006), UPDRS-III (*r* = 0.202, *p* = 0.045), and BMI (*r* = −0.299, *p* = 0.003). In contrast to YM, the correlations of SWV with the UPDRS total score (*r* = 0.197, *p* = 0.051) and male sex (*r* = 0.194, *p* = 0.053) showed a trend toward significance but did not reach the conventional statistical threshold.

**FIGURE 3:**
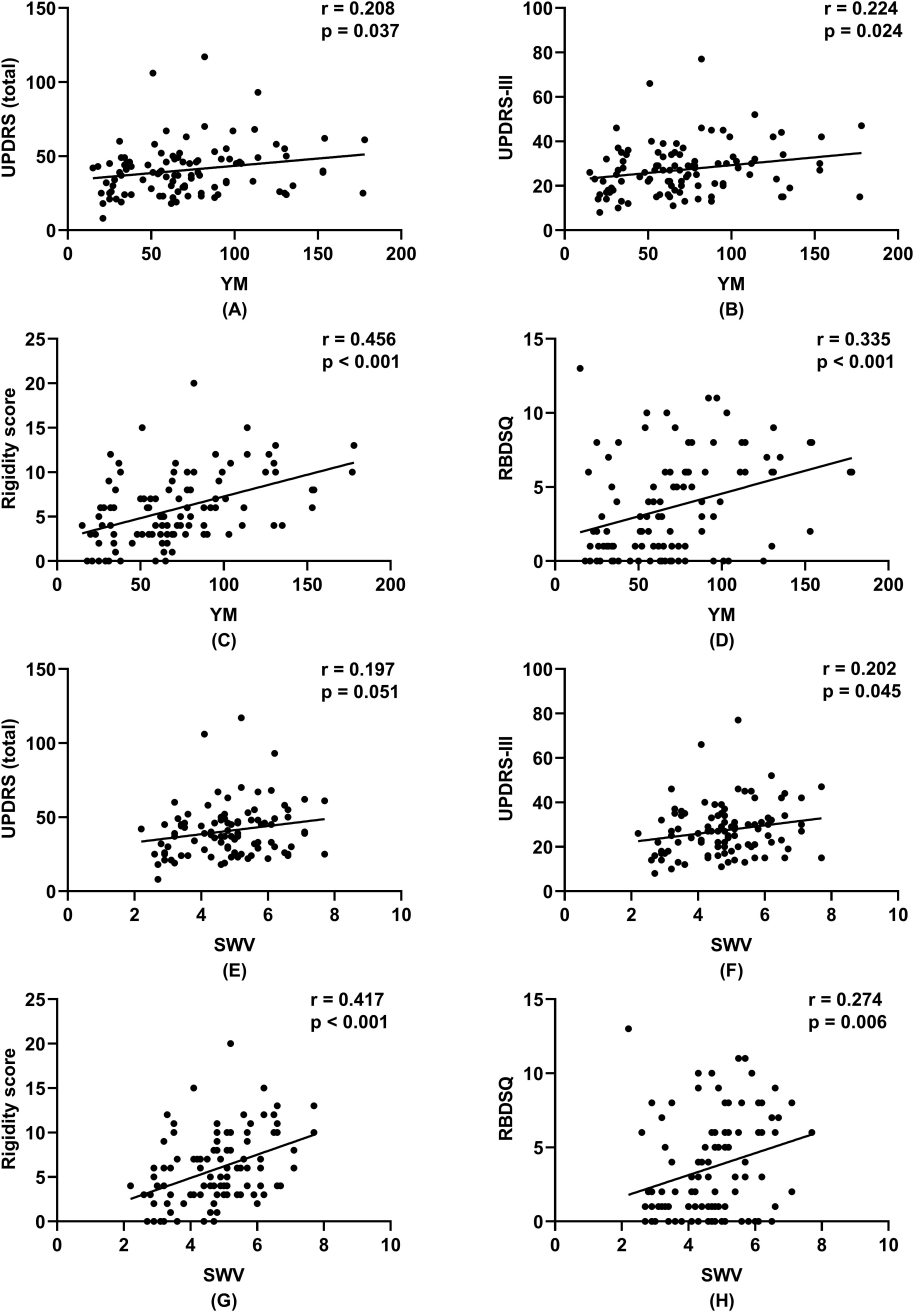
Correlations between shear wave elastography parameters and clinical scores in Parkinson’s disease patients. Scatter plots show the relationship of Young’s modulus (YM) and shear wave velocity (SWV) with **(A,E)** the total Unified Parkinson’s Disease Rating Scale (UPDRS) score, **(B,F)** with UPDRS Part III score, **(C,G)** with rigidity score, and **(D,H)** with the REM sleep behavior disorder screening questionnaire (RBDSQ) score. Pearson correlation coefficients (r) and corresponding *p*-values are provided in each panel.

For both YM and SWV, no significant correlations were detected with age, disease duration, UPDRS-I, UPDRS-II, UPDRS-IV, HAMD, HAMA, NMSQ, MMSE, PDSS, or PDQ-39 (all *p* > 0.05; Figure 3 and Table 3).

**TABLE 3:** Correlation between SWE parameters (YM and SWV) and participant characteristics.

Variable	YM		SWV	
	*R*-values	*P*-values	*R*-values	*P*-values
Male	0.199	0.045	0.194	0.053
BMI	−0.304	0.002	−0.299	0.003
UPDRS	0.208	0.037	0.197	0.051
UPDRS-III	0.224	0.024	0.202	0.045
Rigidity score	0.456	<0.001	0.417	<0.001
RBDSQ	0.335	<0.001	0.274	0.006

Pearson or Spearman correlation coefficients (r) and corresponding *p*-values are shown. SWE, shear wave elastography; YM, Young’s modulus; SWV, shear wave velocity; BMI, body mass index; UPDRS, Unified Parkinson’s Disease Rating Scale; RBDSQ, Rapid Eye Movement sleep behavior disorder screening questionnaire.

To determine whether the association between SWE parameters and PD subtype was independent of BMI and sex, binary logistic regression analyses were performed. After adjusting for BMI and sex, both YM (adjusted Odds Ratio [OR] = 1.029, 95% CI: 1.014–1.044, *p* < 0.001) and SWV (adjusted OR = 1.935, 95% CI: 1.287–2.909, *p* = 0.002) remained significant independent predictors of the body-first subtype.

### 3.5 ROC curve analysis

**FIGURE 4:**
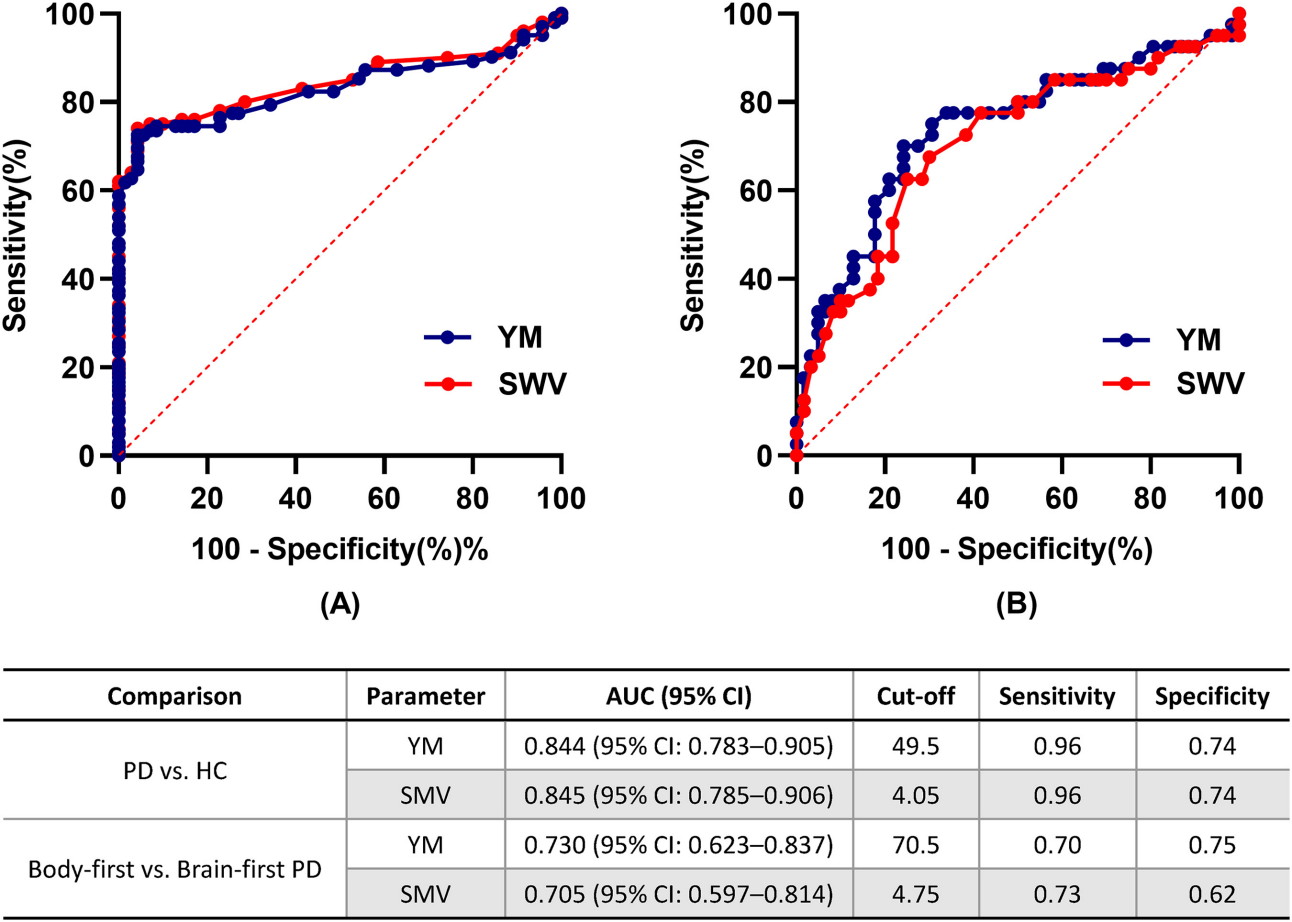
Receiver operating characteristic curves for SWE parameters in discriminating PD from controls and differentiating PD subtypes. **(A)** Young’s modulus (YM) and shear wave velocity (SWV) for distinguishing PD from HC. **(B)** YM and SWV for distinguishing body-first from brain-first PD. The corresponding diagnostic metrics for each comparison are summarized in the table below.

Receiver operating characteristic curves evaluating the exploratory diagnostic performance of YM and SWV are presented in Figure 4. For discriminating PD from controls, YM yielded an AUC of 0.844 (95% CI: 0.783–0.905, *p* < 0.001) with a cut-off of 49.5 kPa (sensitivity = 0.96, specificity = 0.74). SWV produced an AUC of 0.845 (95% CI: 0.785–0.906, *p* < 0.001) with a cut-off of 4.05 m/s (sensitivity = 0.96, specificity = 0.74). In an exploratory analysis for differentiating PD subtypes, YM achieved an AUC of 0.730 (95% CI: 0.623–0.837, *p* < 0.001) at a cut-off of 70.5 kPa (sensitivity = 0.70, specificity = 0.75), whereas SWV showed an AUC of 0.705 (95% CI: 0.597–0.814, *p* = 0.001) at a cut-off of 4.75 m/s (sensitivity = 0.73, specificity = 0.62).

### 3.6 Sensitivity analysis

A sensitivity analysis, in which 14 patients with borderline RBDSQ scores (4 or 5) were excluded, was conducted to assess the robustness of the subtyping approach (Supplementary Table 1). The analysis included 90 PD patients (56 brain-first, 34 body-first) and 68 HCs. The key findings of the primary analysis remained largely unchanged. Specifically, significant differences in YM and SWV persisted among the three groups (all *p* < 0.001 after Bonferroni correction), with the definite body-first subgroup displaying the highest values.

For clinical scales, differences in axial/gait symptoms (*p* = 0.058) and PDQ-39 scores (*p* = 0.053) between the definite body-first and brain-first subgroups were attenuated to a trend level and no longer reached statistical significance. However, all other previously significant clinical distinctions, including tremor (*p* = 0.007), rigidity (*p* = 0.001), and non-motor symptoms (UPDRS-I, HAMD, HAMA, NMSQ, PDSS, RBDSQ; all *p* < 0.05), remained statistically significant. This confirms that the core phenotypic differences between the subtypes are robust to potential misclassification.

## 4 Discussion

This exploratory study employed SWE to quantify biceps brachii stiffness using YM and SWV in HCs and PD patients. We found significantly higher YM and SWV values in PD patients than in HCs. Importantly, subgroup analysis indicated that patients classified as PD exhibited greater YM and SWV than those classified as brain-first PD. These SWE parameters also correlated significantly with several clinical scores. Clinically, compared to the brain-first subgroup, the body-first subgroup presented with a higher burden of non-motor symptoms, more severe rigidity and axial/gait impairment, yet lower tremor scores.

To address potential classification bias, a sensitivity analysis excluding patients with borderline RBD symptoms was performed. This analysis supported the primary findings, showing that the increased muscle stiffness and key clinical features of the body-first subtype remained robust. Finally, multivariate logistic regression confirmed that the association between elevated SWE parameters (YM and SWV) and the body-first subtype persisted after adjusting for confounders such as body mass index and sex, further underscoring the robustness of the relationship.

This study provides the first evidence of a quantifiable difference in muscle biomechanical properties, as measured by SWE, between PD patient groups classified according to current clinical criteria for brain-first and body-first subtypes. The finding of increased stiffness in the body-first group aligns with the hypothesized greater involvement of brainstem and axial motor pathways in this subtype (Asci et al., 2023; Zampogna et al., 2024). However, it is crucial to emphasize that this is a pilot, cross-sectional observation. The clinical subtyping in this study was based on the RBDSQ, a screening tool, rather than the gold-standard PSG. Therefore, these results should be interpreted as preliminary and hypothesis-generating, highlighting a novel biomechanical dimension for further investigation rather than validating SWE as a standalone subtyping tool.

Shear wave elastography has gained significant traction in musculoskeletal research and clinical practice for diagnosing disorders, monitoring progression, and evaluating rehabilitation outcomes (Romano et al., 2021; Flatres et al., 2020; Blank et al., 2022; Playford et al., 2023; Albano et al., 2024). However, its application for investigating muscle stiffness in PD remains limited. Seminal studies by Du et al. (2016) and Ding et al. (2021) established that SWE parameters are elevated in the biceps brachii and brachioradialis muscles of PD patients and correlate with clinical rigidity scores–a core finding robustly replicated in our study. Further work by Ding et al. (2024) in the gastrocnemius revealed differential stiffness changes between PD motor phenotypes post-exercise, while Oppold et al. (2023) demonstrated SWE’s sensitivity to short-term rigidity modulation by medication and deep-brain stimulation.

Our findings align with this literature while clarifying important methodological contexts (Du et al., 2016; Ding et al., 2021). All three primary studies used similar linear-array transducers (frequency range: 4–15 MHz), and both our work and Ding et al. (2021) standardized measurements in the “ON” medication state, whereas Du et al. (2016) did not specify medication status. While Du et al. (2016) focused on the biceps brachii in H–Y I–II patients, our cohort encompassed a broader range (H–Y I–III), potentially enhancing generalizability. Despite these nuances, the convergent evidence firmly establishes SWE as a sensitive tool for quantifying rigidity in PD. Building on this foundation, our study provides a novel extension by applying SWE to explore differences between body-first and brain-first PD groups–a new application beyond prior phenotypic distinctions. This positions SWE as a potential adjunctive tool for enriching the phenotypical characterization of PD subtypes, complementing its established role in quantifying generalized rigidity.

Notably, previous studies reported no significant differences in overall motor symptom severity between these subtypes–a finding consistent with our observation that H–Y stage, total UPDRS, and UPDRS-III scores did not differ substantially between groups (Horsager et al., 2020; Lövdal et al., 2024). This indicates that general motor severity may not reliably differentiate body-first from brain-first PD. However, our results show that body-first PD patients exhibit higher rigidity and axial/gait scores, alongside lower tremor scores. RBD, the second most common sleep disorder in early PD, is strongly associated with the body-first subtype (Dodet et al., 2024). Previous studies suggest that PD patients with RBD are more likely to present with a non-tremor-dominant phenotype (Lee et al., 2010; Liu et al., 2019; Romenets et al., 2012). Moreover, in patients with idiopathic RBD who later develop PD, bradykinesia and rigidity often precede tremor (Fereshtehnejad et al., 2019). This supports the view that the tremor-dominant phenotype may be more characteristic of brain-first PD, while the non-tremor-dominant phenotype may align with body-first PD. The neural mechanisms underlying rigidity and tremor appear distinct, as evidenced by the lack of correlation between their severity (Wu et al., 2023). Furthermore, tremor-dominant patients typically experience slower disease progression than those dominated by rigidity. As a core PD symptom affecting approximately 89% of patients, rigidity is closely linked to reduced dopamine in the basal ganglia (Berardelli et al., 1983; Moustafa et al., 2016), though its precise pathophysiology remains debated. Proposed mechanisms include enhanced monosynaptic and prolonged-latency stretch reflexes, the emergence of tonic stretch reflexes, and shortened response times, as well as decreased neuronal firing in the subthalamic nucleus and altered functional connectivity among cerebellar, motor-cortical, temporal-cortical, occipital-cortical, and caudate networks in moderate PD (Berardelli et al., 1983; Zampogna et al., 2024; van Nuland et al., 2020; Ferreira-Sánchez et al., 2020).

The pathophysiological basis for the pronounced rigidity in body-first PD is likely multifactorial. This subtype is characterized by early α-syn pathology in the brainstem, affecting nuclei such as the locus coeruleus and the magnocellular reticular formation (Borghammer and Van Den Berge, 2019). We hypothesize that this early brainstem involvement may disproportionately disrupt non-dopaminergic pathways, including descending reticulospinal tracts that are crucial for the control of axial and postural tone (Shen et al., 2020). This could manifest clinically as greater rigidity and axial impairment, as observed in our body-first subgroup. Additionally, dysregulation of GABAergic neurotransmission, which plays a role in both motor control and REM-sleep regulation, may constitute a shared mechanism underlying both the prominent RBD and the severe rigidity in this subtype (Al-Kuraishy et al., 2024; van Nuland et al., 2020). Thus, the body-first phenotype may reflect a more widespread brainstem pathology that not only triggers RBD but also primes the motor system for greater stiffness.

A recent review of clinical and imaging evidence supports the association between body-first PD and a higher burden of non-motor symptoms (Horsager et al., 2022). In a comparative study including 65 PD patients and 33 healthy subjects, 46% of those with PD and RBD also exhibited symptoms of apathy and depression (Barber et al., 2018). Consistent with these reports, our results demonstrated a greater non-motor symptom burden in the body-first subgroup, as reflected by elevated scores across multiple PD assessment tools, including UPDRS-I, HAMD, HAMA, NMSQ, RBDSQ, PDSS, and PDQ-39. Although it has been hypothesized that brain-first PD, characterized by initial α-syn aggregation in the brain, might confer a higher risk of cognitive impairment or dementia, this view is not universally supported. A systematic review indicated that sleep disturbances–particularly RBD–are correlated with cognitive dysfunction (Maggi et al., 2021). PD patients with RBD tend to exhibit more significant cognitive deficits and a faster progression to dementia. This may be linked to reduced neuromelanin in the locus coeruleus, decreased noradrenergic transporter density, and diminished cortical acetylcholinesterase activity, all markers associated with poorer cognitive performance (Sommerauer et al., 2018; Kotagal et al., 2012). These findings suggest that body-first PD could have a more substantial impact on cognition than the brain-first subtype. However, cognitive function is influenced by a complex interplay of neurochemical systems and biological mechanisms, making it challenging to clearly differentiate between PD subtypes based solely on cognitive profiles. In the present study, we observed no significant differences in MMSE scores between the two subtypes.

In analyzing the correlation between SWE parameters and participant characteristics, we observed significant associations with rigidity score, UPDRS total, UPDRS-III, RBDSQ, male sex, and BMI. These results align with previous studies, which also reported positive correlations between SWE-derived parameters and UPDRS scores (Du et al., 2016; Ding et al., 2021). Existing literature shows divergent conclusions regarding the association between muscle stiffness and demographic factors such as age, sex, and BMI (Creze et al., 2018; Chodock et al., 2020; Blank et al., 2022). The observed weak negative correlation between SWE metrics and BMI in our cohort aligns with a growing body of literature on body composition’s influence on muscle-stiffness measurements. This relationship appears to be muscle-specific, as evidenced by reports of both negative correlations in the biceps brachii and neck muscles (Chodock et al., 2020; Kocur et al., 2019; Kuo et al., 2013), and positive correlations in other muscles such as the genioglossus (Ichikawa et al., 2024). Most pertinently, our finding is consistent with a recent study by Nakano et al. (2025), which utilized an objective rigidity instrument and similarly reported a significant association between higher BMI and lower objectively measured rigidity. A unifying physiological explanation for the negative correlation observed in limb muscles is the impact of adipose tissue. Higher BMI often correlates with greater intramuscular fat infiltration (myosteatosis). Because shear waves propagate more slowly through softer adipose tissue than through dense muscle fibers, increased fat content within the biceps brachii at higher BMI likely reduces the overall composite stiffness measured by SWE. This insight is crucial for clinical interpretation: SWE quantifies the composite stiffness of the tissue, which reflects both the neurogenic component of rigidity and the intrinsic mechanical properties of the muscle structure itself, with body composition being an important consideration when comparing absolute values across individuals. The effect of age on SWE measurements remains controversial, with some studies indicating an increase in SWV with age (Liu et al., 2021; Eby et al., 2015) and others a decrease (Şendur et al., 2020; Akagi et al., 2015). Wang et al. (2021) observed significantly higher SWV in the middle deltoid muscle among males compared with females. In line with this, we identified a weak positive correlation between YM and male sex but no significant association with age. Although statistically significant weak correlations were observed between SWE parameters and factors such as BMI and sex, their clinical significance is limited. These associations highlight the importance of considering body composition and sex as potential covariates in future studies aiming to standardize SWE measurements across diverse populations.

We found significantly higher YM and SWV values in body-first PD, with moderate diagnostic accuracy for subtyping (AUC ≈ 0.7). This level of accuracy, while not sufficient for standalone clinical diagnosis, positions SWE as a potential adjunctive or screening tool in research settings. It could be valuable for enriching research cohorts or contributing to a multimodal subtyping algorithm in conjunction with established biomarkers. The practical utility of SWE must be positioned appropriately relative to established biomarkers.

Advanced imaging techniques, including PET, ^123^I-MIBG myocardial scintigraphy, and striatal dopaminergic imaging, have proven valuable in differentiating PD subtypes (Horsager et al., 2020; Park et al., 2024; Knudsen et al., 2021). SWE does not aim to replace these modalities. Instead, it offers a complementary and novel perspective by quantifying a direct biomechanical consequence of the disease–increased rigidity. As a non-invasive, radiation-free, and readily accessible point-of-care technique, SWE provides a unique value proposition. Our study, by being the first to report SWE-measured stiffness differences between groups classified as the body-first and brain-first subtypes, provides novel insights into the biomechanical distinctions between these subgroups and highlights SWE’s promise as an exploratory tool in the broader investigation of PD heterogeneity.

Several methodological considerations and future directions emerge from our study. First, our selection of the mean values (YM and SWV) as the primary SWE parameters was physiologically and clinically motivated. The mean provides a stable, reproducible measure of the global muscle stiffness within the region of interest, reflecting the overall biomechanical state. In contrast, maximum values are susceptible to local heterogeneity or artifacts, and minimum values may underestimate pathological tone, making the mean the most representative metric for generalized rigidity. Second, the influence of dopaminergic medication must be considered. All SWE measurements were performed in the practical “ON” medication state to standardize patient condition and cooperation. While this reflects the typical treated state, it likely led to an underestimation of the true baseline differences in muscle stiffness between subtypes, as levodopa ameliorates rigidity. Future studies incorporating “OFF”-state assessments are essential to establish unmedicated biomechanical properties. Moreover, repeated SWE across the medication cycle could transform it into a dynamic biomarker, quantifying the rigidity response to levodopa and potentially revealing subtype-specific dopaminergic modulation patterns.

The potential ability to distinguish between body-first and brain-first PD subtypes holds significant clinical implications. Identifying the subtype at diagnosis could provide valuable prognostic information, as body-first PD is often associated with a more rapid progression of specific non-motor symptoms and possibly a different trajectory of motor decline (Horsager et al., 2022; Dong et al., 2024; Xu et al., 2024). This understanding could enable clinicians to manage patient expectations more effectively and implement a more personalized and proactive management strategy. For instance, patients classified as body-first PD might benefit from earlier and more aggressive management of non-motor features such as RBD, autonomic dysfunction, and mood disorders. Furthermore, this subtyping has profound implications for clinical-trial design, ensuring more homogeneous patient cohorts and thereby enhancing the sensitivity to detect subtype-specific therapeutic responses. The observation that SWE can detect biomechanical differences between these groups adds a novel, quantifiable layer to their characterization, which could support these critical clinical distinctions in future, validated multimodal frameworks.

Looking forward, our work suggests several key research pathways. Longitudinally, SWE could track rigidity progression within subtypes. Therapeutically, it may objectively monitor responses to pharmacologic and physical interventions. Most powerfully, future studies should integrate SWE into a multimodal subtyping framework, combining it with gold-standard clinical scales, PSG-confirmed RBD status, MIBG scintigraphy, and dopaminergic imaging to construct a composite biomarker profile for mechanism-based stratification and personalized management. This is essential to validate the role of SWE-measured biomechanical properties in PD subtyping.

This study has several limitations. First, the classification of PD subtypes relied on the RBDSQ rather than PSG, which may introduce misclassification bias. This is the most significant limitation, and it means our findings are preliminary. A sensitivity analysis excluding borderline RBDSQ scores confirmed the robustness of our core SWE findings, though differences in axial symptoms and quality of life were attenuated, suggesting these features may be more sensitive to classification criteria. Second, all patients were at H–Y stages I–III, limiting generalizability to advanced PD. Third, the lack of biopsy or MRI data precluded correlation of SWE measures with underlying neuropathology. Finally, as noted above, the exclusive use of the “ON” medication state may have obscured the full extent of biomechanical differences between subtypes. Crucially, SWE measures a downstream biomechanical property (muscle stiffness) rather than the site of pathological onset, which is the defining feature of the brain-first/body-first hypothesis. Therefore, our study demonstrates an association, not a validation of SWE for subtyping. Future studies including OFF-state assessments and PSG-confirmed cohorts are warranted to validate and extend our findings.

## 5 Conclusion

In summary, our findings demonstrate that SWE is a sensitive and non-invasive ultrasound technique for quantifying muscle rigidity in PD. Moreover, by revealing increased rigidity in the group classified as body-first compared to the group classified as brain-first subtype, our study identifies a novel biomechanical difference between these PD subgroups. These pilot results suggest that SWE-measured muscle stiffness may represent a new phenotypic dimension in PD. However, the moderate discriminatory accuracy and the reliance on questionnaire-based subtyping preclude its use as a standalone diagnostic tool. Future work must integrate SWE with established clinical and imaging biomarkers in longitudinal, PSG-confirmed cohorts to determine its potential utility as a complementary metric for refining PD subtype classification and advancing toward more personalized management strategies.

## Data Availability

The raw data supporting the conclusions of this article will be made available by the authors, without undue reservation.
