# Supplementary material for: Exploring biomechanical differences between brain-first and body-first Parkinson’s disease subtypes using shear wave elastography: a pilot cross-sectional study
Source: Front Aging Neurosci. 2026 Feb 2;18:1706502. doi: 10.3389/fnagi.2026.1706502 (PMC12907343; doi:10.3389/fnagi.2026.1706502)
Supplement: Supplementary file 1 [file Table_1.docx]

**Supplementary TABLE S1 Sensitivity Analysis Excluding Borderline RBDSQ Scores: Comparison of demographic, clinical characteristics, and SWE parameters among healthy controls, brain-first PD, and body-first PD subgroups.**

|  | **HC (n = 68)** | **PD (n = 90)** | | **p-values** |
| --- | --- | --- | --- | --- |
|  |  | Brain-first PD (n = 56) | Body-first PD (n = 34) |  |
| General information |  | | | |
| Age (years) | 64.96 ± 8.55 | 66.63 ± 9.33 | 68.26 ± 7.68 | 0.181 |
| Sex (male, N%) | 26 (38.24) | 26(46.43) | 20 (58.82) | 0.142 |
| BMI (kg/m^2^) | 23.60 ± 3.37 | 24.34 ± 4.08 | 23.85 ± 3.66 | 0.543 |
| Hypertension (N%) | 27 (39.71) | 23 (41.07) | 18 (52.94) | 0.416 |
| SWE ultrasound |  | | | |
| YM(KPa) | 33.56 ± 8.44 | 58.16 ± 29.16^a^ | 92.82 ± 42.47^ab^ | <0.001 |
| SWV (m/s) | 3.31 ± 0.41 | 4.41 ± 1.11^a^ | 5.39 ± 1.39^ab^ | <0.001 |
| Disease duration (years) | -- | 3.00 (1.00, 4.00) | 2.25 (1.00, 4.00) | 0.638 |
| Assessment scales |  | | | |
| Hoehn & Yahr stage | -- | 2.39 ± 0.55 | 2.54 ± 0.64 | 0.235 |
| UPDRS (total) | -- | 37.44 ± 15.51 | 42.44 ± 20.24 | 0.192 |
| UPDRS-I | -- | 1.00 (0.00, 2.00) | 2.00 (1.00, 3.00)^b^ | 0.029 |
| UPDRS-II | -- | 10.04 ± 5.39 | 11.18 ± 7.53 | 0.408 |
| UPDRS-III | -- | 25.53 ± 11.07 | 28.50 ± 12.82 | 0.250 |
| Tremor score | -- | 4.15 ± 4.09 | 2.24 ± 2.38^b^ | 0.007 |
| Rigidity score | -- | 4.75 ± 3.61 | 7.47 ± 4.05^b^ | 0.001 |
| Bradykinesia score | -- | 10.73 ± 5.86 | 12.35 ± 7.22 | 0.248 |
| Axial/Gait score | -- | 3.38 ± 1.58 | 4.06 ± 1.67 | 0.058 |
| UPDRS-IV | -- | 0.00 ( 0.00, 0.00) | 0.00 (0.00, 0.00) | 0.216 |
| HAMD | -- | 5.76 ± 4.57 | 8.79 ± 7.47^b^ | 0.038 |
| HAMA | -- | 4.33 ± 3.60 | 6.59 ± 5.43^b^ | 0.036 |
| NMSQ | -- | 7.15 ± 4.39 | 9.24± 5.03^b^ | 0.042 |
| PDSS | -- | 121.56 ± 20.59 | 110.06 ± 19.64^b^ | 0.011 |
| RBDSQ | 1.24 ± 1.02 | 1.00 ± 1.04 | 7.74± 1.83^ab^ | <0.001 |
| MMSE | 28.00 (26.00, 29.75) | 27.00 (25.00, 28.00)^a^ | 27.00 (24.00, 29.25)^a^ | 0.049 |
| PDQ39 | -- | 25.19 ± 19.51 | 34.71± 25.96 | 0.053 |

Data are expressed as mean ± SD, median (IQR), or count (%). A Bonferroni correction was applied for SWE parameters (2 parameters × 3 comparisons; significance threshold: p < 0.0083). Superscripts indicate pairwise differences: a vs. HC group; b vs. brain-first PD group. Abbreviations: see Table 1 and Table 2.
